# Impact of Initial Cyclic Loading on Mechanical Properties and Performance of Nafion

**DOI:** 10.3390/s23031488

**Published:** 2023-01-29

**Authors:** David Vokoun, Sneha Samal, Ivo Stachiv

**Affiliations:** Department of Functional Materials, Institute of Physics, Czech Academy of Sciences, Na Slovance 2, 18221 Prague, Czech Republic

**Keywords:** Nafion, mechanical tests, viscoplastic properties, cyclic loading, mechanical properties

## Abstract

Nafion possesses many interesting properties such as a high ion-conductivity, hydrophilicity, and thermal and chemical stability that make this material highly suitable for many applications including fuel cells and various (bio-)chemical and physical sensors. However, the mechanical properties of a Nafion membrane that are known to be affected by the viscoplastic characteristics of the material itself have a strong impact on the performance of Nafion-based sensors. In this study, the mechanical properties of Nafion under the cyclic loading have been investigated in detail. After cyclic tensile loading (i.e., maximum elongation about 25% at a room temperature and relative humidity about 40%) a time-dependent recovery comes into play. This recovery process is also shown being strain-rate dependent. Our results reveal that the recovery behavior weakens after performing several stress–strain cycles. Present findings can be of a great importance in future design of various chemical and biological microsensors and nanosensors such as hydrogen or glucose ones.

## 1. Introduction

Nafion, a brand name for a sulfonated tetrafluoroethylene-based fluoropolymer-copolymer, has attracted a great attention since its discovery in the late 1960s. The interest in Nafion is derived from its unique structure [1,2] and properties (i.e., good chemical and thermal stability, high hydrophilicity of ionic-water domains, and ion-conductivity to cations, as well as permeability to water, etc.); there it is used in various models [3] and applications ranging from various sensors [4,5,6,7,8,9,10,11,12,13,14,15], fuel cells [16], and water electrolyzers [17] to chlor-alkali cells [18]. In addition, Nafion is the key constituent in the ionic polymer–metal composites (IPMCs) working as sensors and actuators [19,20]. However, most Nafion applications relate to Nafion’s sensor capabilities. It is worth of noting that indispensable part of Nafion-based microsensor/nanosensor development is the understanding of mechanics of the sensor material. Hence, to enhance the sensitivity and reliability of the Nafion-based sensors, the dependency of the fundamental mechanical properties of Nafion (e.g., the Young’s modulus and the Poisson’s ratio) on the various external conditions must be known [16,19]. As for mechanical properties, the character of tensile stress–strain response is also of a great importance. The mechanical properties and structure of Nafion depend on temperature, the level of hydration, the previous loading history [21,22,23,24,25], and thickness of the Nafion (see Ref. [26] for Nafion in the form of thin film and Refs. [27,28] for a bulk membranes). Modeling of the mechanical properties of Nafion takes into account various factors affecting the behavior of Nafion, such as its membrane structure [29], morphology [30], thermodynamic activity of water [31], and/or its viscoelastic/viscoplastic character [23,32,33]. For example, the dependence of Young’s modulus of Nafion on the hydration level was discussed by Nemat-Nasser et al. [34,35]. The Poisson’s ratio of the Nafion membranes was reported to be around 0.4 in ambient conditions [36]. It has also been shown that the Poisson’s ratio of the Nafion increases with increasing fraction of uncompressible water molecules [37]. Then, the dependency of the mechanical properties of the “Nafion 117” membrane on temperature and humidity was evaluated by Bauer et al. [38]. Similarly, the effect of cyclic loading on fatigue properties of Nafion membrane was also studied [39]. However, in these studies the authors considered many thermomechanical cycles without focusing on the initial several loading cycles.

In this work, an experimental investigation on Nafion under cyclic loading is performed. Unlike the cyclic loading study in [39], the present cyclic loading study involves only several initial loading cycles. Beside the stress–strain behavior, the results of conventional thermal analysis such as differential scanning calorimetry and thermodynamic analysis are presented. As for stress–strain behavior, stress-free recovery as a function of the strain rate has been studied. The origin of the recovery was elucidated to residual stresses established after loading and following unloading. We remind the reader that the shape memory phenomena were already reported [40]. Briefly, the temperature memory effect refers to the capability of Nafion to memorize temperature at which the deformation of Nafion sample occurs. After deformation, the phenomenon was observed through a recovery stress increase when varying temperature at the iso-strain condition (strain kept constant) [40]. Importantly, the strain recovery observed in our study bears some similarities with the pseudoelastic effect of shape memory alloys [41] loaded above temperature Af (austenite finish). The pseudoelastic recovery takes place at a constant temperature; however unlike Nafion recovery, the pseudoelastic recovery proceeds immediately during unloading. In addition, the main differences between the shape memory phenomena observed in both the shape memory alloys and Nafion (i.e., in the present study the Nafion in the form of 180 µm thick membrane is considered) are shown. Finally, the possible cause of the behavior as well as the application of Nafion ultra-thin foils in design of micro-/nanosensors is discussed.

## 2. Materials and Methods

**Figure 1:**
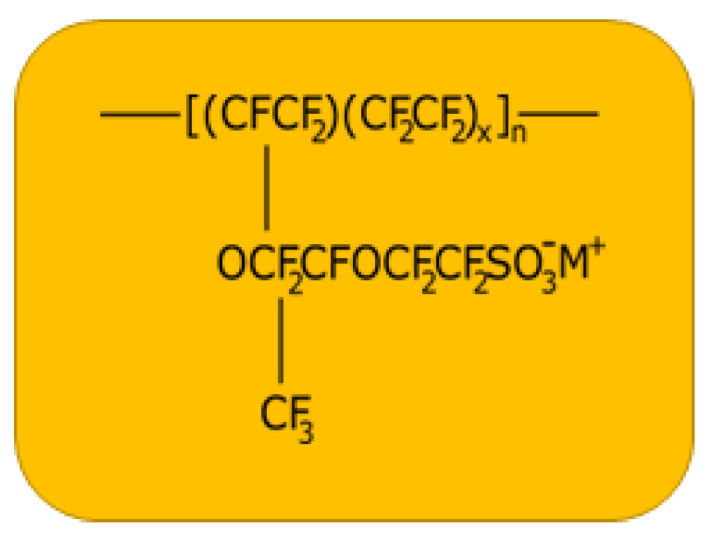
The chemical structure of the used Nafion membrane-N117 where x = 6.5, and M^+^ stands for an exchangeable counterion.

A commercial Nafion membrane N117 of thickness about 180 µm was purchased from IonPower company. The structure of commercially available Nafion-117 membrane is shown in Figure 1. The used symbols are *x* = 6.5, and M^+^ is the exchangeable counterion with a capacity of 0.91 meq g^−1^ (equivalent weight = 1100).

All the experiments were carried out in ambient temperature with a stable relative humidity of ~40%. The transition temperatures were obtained using the differential scanning calorimeter (DSC), (TA Instruments Trios v5.1.1.46572, New Castle, DE, USA). The thermal rate during heating and cooling was 5 K/min. The used gas in the DSC chamber was N_2_ with the gas flow 60 mL/min. Thermodynamic analysis was conducted using a DMA analyser (DMA 850 tester from TA Instruments). The fixed frequency was 1 Hz, and the temperature range was from 30 °C to 120 °C. The loss and storage modulus and tan(delta) (internal friction) were evaluated in the temperature range. The mechanical tests were caried out at room temperature on thermo-mechanical tester (Walter + Bay), described in Ref. [42], with various strain rates. The sample stretching in the tester was controlled by engineering strain.

## 3. Results and Discussion

**Figure 2:**
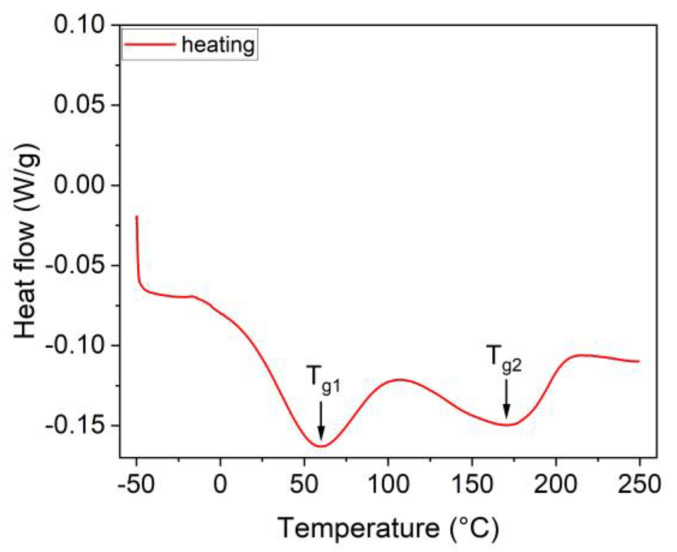
Dependency of heat flow on temperature used for determination of the transition temperatures.

The dependency of heat flow on temperature is presented in Figure 2. As can be seen from the results, two endothermic peaks at 60 °C and 170 °C during heating the sample were observed. Both of these peaks marked as T_g1_ and T_g2_ represent the glass transitions. According to Ref. [43], T_g1_ and T_g2_ peaks might be related to the change of mobility of the Nafion’s main chain and to the side chain due to the interactions among the sulfonic acid functional groups, respectively.

**Figure 3:**
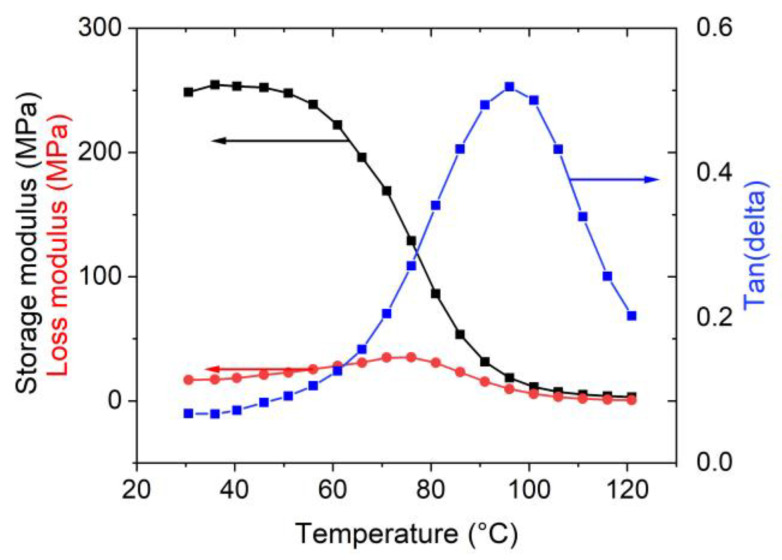
The diagram of storage and loss moduli, and internal friction as a function of temperature from 30 °C to 120 °C.

Figure 3 shows the dependency of the storage and loss moduli and tan(delta) on temperature which ranges from 30 °C to 120 °C. The onset of the storage modulus, which decreases during the sample heating, corresponds to the polymer softening at temperature of 60 °C (see Figure 2). The increase in tan(delta) during heating up after exceeding T_g1_ is related to the internal friction in the membrane.

**Figure 4:**
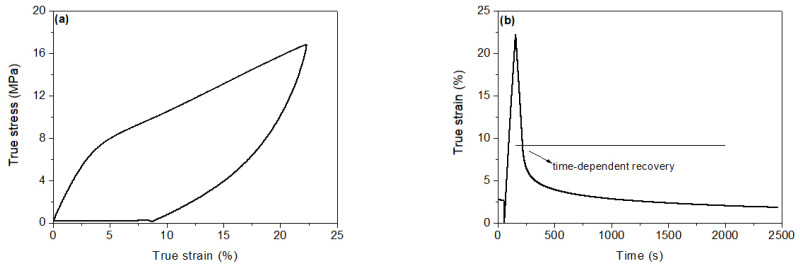
(**a**) Relationship between true stress and true strain and (**b**) Observed dependency of the strain recovery on time.

Finally, the effect of cyclic loading on the mechanical properties of Nafion which is needed for the successful design of the various Nafion-based microsensors/nanosensors was evaluated by performing a series of mechanical tests. At the beginning, the first strain recovery at room temperature after one loading cycle was observed. Figure 4a shows diagram of true stress versus true strain for a strain rate of 0.25%/s. The dependency of strain recovery on time is given in Figure 4b.

It is noteworthy that a significant strain recovery after unloading can be observed. Briefly, the strain returns from level 7.8% to 2.4% under a small load (i.e., the small load of 0.3 N keeps the sample stretched in order to facilitate strain reading in the measurement apparatus) in 40 min. Then, in addition to this time-dependent strain recovery, during unloading a large part of strain is also recovered, that is, from 22% to 7.8%.

**Figure 5:**
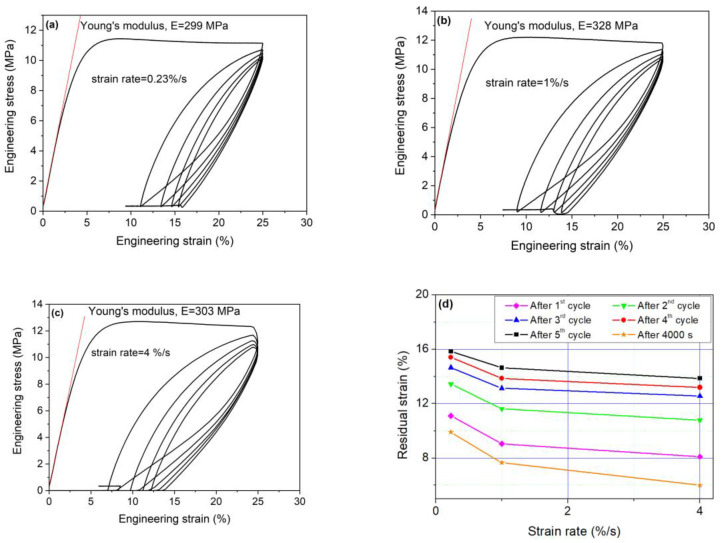
Relationship between the engineering stress and strain for strain rate of (**a**) 0.23%/s, (**b**) 1%/s and (**c**) 4%/s. The linear slope of the linear part (red color line) stands for the Young’s modulus and (**d**) residual strain summary for various strain rates.

**Figure 6:**
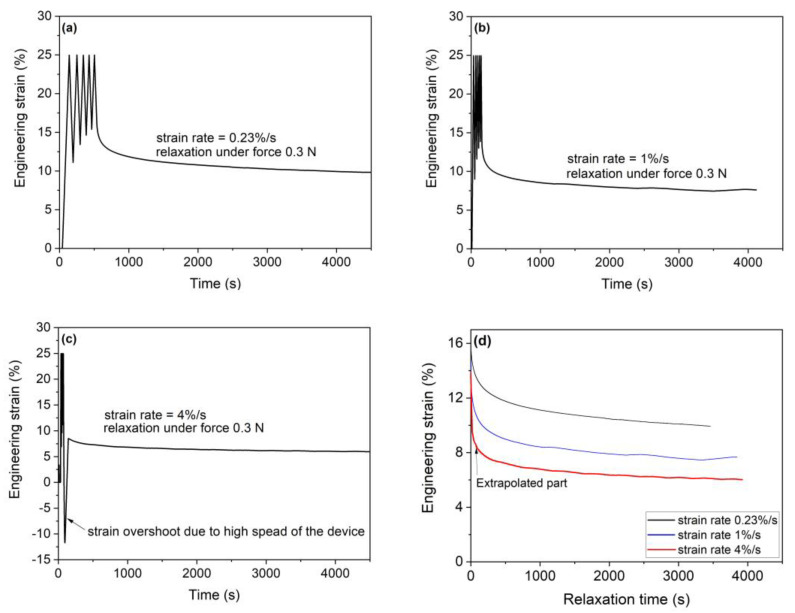
Dependence of strain recovery on time strain rate of (**a**) 0.23%/s, (**b**) 1%/s and (**c**) 4 %/s and (**d**) strain recovery versus relaxing time for all the test strain rates.

We emphasize here that for the sensing purposes, it is necessary to understand how the strain recovery depends on strain rate for the especially several loading cycles. Similarly, it is also necessary to evaluate whether the Young’s modulus of Nafion is strain rate dependent. We perform the cyclic loading, and the obtained experimental results are summarized in Figure 5 and Figure 6. The dependency of the engineering stress on engineering strain for various strain rates (i.e., strain rates were 0.23%/s, 1%/s and 4%/s, respectively) and five loading cycles is given in Figure 5. Figure 6 presents the engineering strain recovery versus time after final (i.e., fifth) unloading. Finally, the Young’s modulus of Nafion sheet obtained from the red tangent line at the starting linear part of the loading is also given in Figure 5.

## 4. Conclusions

Based on the experimental results given in Figure 5 and Figure 6, the following conclusion can be drawn:

(1) The Young’s modulus does not depend on the strain rate; that is, the present study considered Nafion sheet samples loaded with strain rates of 0.23, 1 and 4%/s, from which the obtained Young’s moduli were approximately identical within the measurement error;
(2) The strain recovery during unloading in the first cycle depends on the speed of strain rate. Briefly, to achieve the larger unloading strain recovery requires the faster strain rate;
(3) With an increasing number of loading cycles, the dependence of strain recovery on the speed of strain rate decreases. Namely, the dependency of strain recovery on the strain rate diminishes with the number of loading cycles. We also show that for all study considered cases the strain recovery is decreases from 25% (first cycle) to about ~15% (final cycle);
(4) As for a time-dependent strain recovery after unloading (i.e., in the fifth cycle), the amount of strain recovery strongly depends on the strain rate. The faster the strain rate is, then the larger strain recovery is. For all the strain rates, the strain recovery slows down with increasing time and is negligible after 4000 s. Besides, the time-dependent strain recovery is due to the viscoplastic character of the polymer and residual stresses established after loading (see modeling with the viscoplasticity theory-based overstress [44,45]). The recovery is not complete due to some damage of the material structure.

Interestingly, our results can be used to either evaluate defects in Nafion-based sensors or can be used to design the humidity nanosensors. It means that for humidity sensors, the hydration affects the heat flow (see Figure 2 and Figure 3), and, correspondingly, it enables an easily accessible determination of a relative humidity, particularly in cases of biosensors operating in liquid environments. We foresee that due to the similarities of Nafion with the shape memory alloys (i.e., the shape memory effect and pseudoelasticity) [46], the Nafion will also be a suitable candidate in design of nanomechanical-based mass spectrometers [47] or various metamaterials [48] such as those considered for biomedical and energy applications.

## Data Availability

Not applicable.
